# [Retracted] Intracisternal administration of an interleukin‑6 receptor antagonist attenuates surgery‑induced cognitive impairment by inhibition of neuroinflammatory responses in aged rats

**DOI:** 10.3892/etm.2023.12333

**Published:** 2023-12-05

**Authors:** Peng Jiang, Qiong Ling, Hongbo Liu, Weifeng Tu

Exp Ther Med 9:982–986, 2015; DOI: 10.3892/etm.2014.2149

Following the publication of this paper, it was drawn to the Editors’ attention by a concerned reader that the western blotting data shown in Fig. 4 were strikingly similar to data appearing in different form in other articles written by different authors at different research institutes that had either already been published elsewhere prior to the submission of this paper to *Experimental and Therapeutic Medicine*, or were under consideration for publication at around the same time. In view of the fact that certain of these data had already apparently been published previously, the Editor of *Experimental and Therapeutic Medicine* has decided that this paper should be retracted from the Journal. The authors were asked for an explanation to account for these concerns, but the Editorial Office did not receive a reply. The Editor apologizes to the readership for any inconvenience caused.

